# The Effects of Acupuncture on Pregnancy Outcomes of Recurrent Implantation Failure: A Systematic Review and Meta-Analysis

**DOI:** 10.1155/2021/6661235

**Published:** 2021-02-02

**Authors:** Menglin Li, Yunyun Liu, Haoran Wang, Shuzhen Zheng, Yinhe Deng, Yuemei Li

**Affiliations:** ^1^Guangzhou University of Chinese Medicine, Guangzhou, Guangdong, China; ^2^The Third Affiliated Hospital of Sun Yat-Sen University, Guangzhou, Guangdong, China; ^3^The First Affiliated Hospital of Guangzhou University of Chinese Medicine, Guangzhou, Guangdong, China

## Abstract

**Objective:**

To systematically evaluate the efficacy and safety of acupuncture for patients with recurrent implantation failure (RIF) undergoing in vitro fertilization-embryo transfer (IVF-ET) and hopefully provide reliable guidance for clinicians and patients.

**Methods:**

Through searching domestic and foreign medical journals, the literature of randomized controlled trials (RCTs) of acupuncture for RIF undergoing IVF-ET was collected. RevMan 5.3 software was used for meta-analysis and Cochrane's risk of bias assessment tool was used to evaluate the quality of the included studies.

**Results:**

Seven documents meeting the criteria were finally included. The results showed that the intervention group contributes more in outcomes including clinical pregnancy rate (RR = 1.90, 95% CI (1.51, 2.40), *P* < 0.05), biochemical pregnancy rate (RR = 1.59, 95% CI (1.27, 1.99), *P* < 0.05), embryo implantation rate (RR = 1.89, 95% CI (1.47, 2.45), *P* < 0.05), and endometrial thickness (MD = 1.11, 95% CI (0.59, 1.63), *P* < 0.05) when compared with the control group, and the difference is statistically significant. In terms of the number of embryo transfers and the type of endometrium, the difference between the acupuncture group and the control group was not statistically significant.

**Conclusion:**

Acupuncture therapy on patients with RIF can improve the pregnancy outcome of patients. It is a relatively effective treatment with satisfactory safety and suitable for clinical application. However, as the quality of the included studies is not good enough, the conclusion of this meta-analysis should be treated with caution. More double-blind RCTs equipped with high quality and large samples are expected for the improvement of the level of evidence.

## 1. Introduction

The occurrence and development of assisted reproductive technology (ART) is a major advancement in the history of human reproduction. Although in vitro fertilization-embryo transfer (IVF-ET) treatment outcomes have advanced dramatically in terms of conception, implantation, and live birth rates, cycles with unexplained recurrent implantation failure (RIF) remain an unsolved and extremely challenging issue. [1] As reported by the European Society of Human Reproduction and Embryology (ESHRE) in 2016, the current clinical pregnancy rate of IVF-ET patients is 33.8%, among which RIF may be one of the important reasons affecting the success rate of IVF-ET pregnancy. There are reports indicating that the incidence of RIF in patients undergoing IVF-ET assisted pregnancy could reach 5% to 10% [2]. However, the exact definition of this entity remains unclear from author to author. Polanski et al. [3] advised current descriptions incorporate the number of previously failed cycles and whether these were fresh or frozen embryos, the number of embryos transferred, and/or their respective quality, or a combination of these factors. Coughlan et al. [4] had defined RIF as the failure to achieve a clinical pregnancy after the transfer of at least four good-quality embryos within a minimum of three fresh or frozen cycles under 40 years of age. Nowadays, RIF is defined as the failure to achieve clinical pregnancy after the transfer of four or more good-quality embryos, which is widely accepted in clinical work [5].

There are many reasons for RIF, mainly related to endometrial pathology, embryo quality, immune factors, and so on. One article [6] analyzed the etiology of RIF in detail, which believed that (1) poor embryo quality is closely related to egg and sperm cells. Among them, a quantitative decrease in the ovarian reserve and qualitative changes in oocyte competence are the problems of eggs, while the main problem of sperm is DNA damage. One study pointed out that postfertilization molecular mechanisms might result in a genetically abnormal embryo [7]. (2) Uterine diseases include endometrial diseases and myometrial diseases, while endometrial diseases specifically include polyps, submucosal fibroids, intrauterine synechiae, chronic endometritis, endometrial microbiota, unexplained thin endometrium, and fluid in the endometrial cavity，and myometrial diseases include uterine fibroids larger than 4 cm, mediastinal uterus, and other diseases. (3) In terms of endocrine factors, one study reported that thyroid ATA positivity might have negative effects on miscarriage and live birth rates [8]. Polycystic ovary syndrome (PCOS) patients have high concentrations of follicular phase luteinizing hormone, insulin resistance, and high leptin concentrations, which may increase miscarriage and decrease implantation rates. Apart from that, uncontrolled diabetes mellitus and elevated levels of HbA1c in the early stages of pregnancy are also related to risks of fetal mortality and spontaneous abortion. (4) Immunology, thrombophilia, and other unexplained recurrent implantation failure are among the factors.

After the treatment of the above diseases that cause RIF, there are still a considerable number of patients who still cannot successfully conceive. Some scholars have studied the use of acupuncture as an auxiliary means to intervene in the treatment of RIF patients. Acupuncture, as an exogenous and natural treatment method, has a comprehensive multichannel, multilink, and multitarget treatment effect. It is the main treatment method in ancient East Asia. However, the therapeutic mechanism of acupuncture for RIF patients is not clear. According to the relevant literature of this search, most think that acupuncture acts on the ‘hypothalamus-pituitary-ovarian' gonadal axis and plays two-way regulation of the gonadal axis. At present, the curative effect of acupuncture is mainly concentrated in the clinical observation stage. There was a meta-analysis [9] of 1,441 ovulation-disorders women showing that the use of acupuncture as a monotherapy significantly improved the rate of pregnancy compared with the use of Clomiphene Citrate (CC) alone. Other researchers [10], using meta-analysis to explore the relationship between acupuncture and patients with low ovarian response (POR), found that acupuncture may improve CPR, AMH, AFC, and the number of retrieved oocytes in women with POR undergoing IVF. Based on the above review of the efficacy of acupuncture on infertility, we have made a hypothesis whether acupuncture could be effective in patients with RIF or not. In view of the fact that there are not much literature on the observation of acupuncture intervention on RIF patients, we hope to objectively evaluate the efficacy of acupuncture on RIF through the analysis. This may be not only a data processing and analysis, but also an exploration of more possibilities of RIF treatment, a little effort and attempt to seek pregnancy hope for women who suffer from infertility.

## 2. Materials and Methods

### 2.1. Inclusion and Exclusion Criteria

The review was carried out following Cochrane and Preferred Reporting Items for Systematic Reviews and meta-analysis (PRISMA) guidelines. [11] Studies involved were supposed to meet the following criteria. (1) Study object: as we have discussed above, because the definition itself is subject to debate, we have cited the most common diagnostic criteria according to the current situation of clinical work. And the diagnostic criteria adopted were the absence of implantation after transfer of cumulatively four good-quality cleavage stages or blastocyst embryos (at most two embryos for each transfer) within two fresh or frozen cycles diagnosed by a negative serum human chorionic gonadotrophin test 14 days after embryo transfer. (2) Study design: the studies were included if they explored acupuncture's effect on RIF pregnant rate. And the methods of acupoint stimulation included not only manual acupuncture but also electroacupuncture, moxibustion, transcutaneous electrical acupoint stimulation, acupoint embedding, etc. Only randomized controlled trials (RCTs) were eligible for inclusion. (3) Outcome indicators: clinical pregnancy rate was a necessary outcome indicator. Other indicators included sex hormone level, number of eggs retrieved, number of fertilized eggs, excellent embryo (assessing as Grade I embryos with uniform embryonic division and without cell debris) rate, endometrial thickness, endometrial type, and biochemical pregnancy rate. Studies that had any of the following criteria were excluded: (1) animal experiment papers, case reports, expert experience, or documents where data is not available; (2) papers with duplicate or identical data; (3) documents with high publication bias; (4) other intervention methods in the treatment group in addition to acupoint stimulation like acupuncture and moxibustion treatment; (5) no required outcome indicators in the research.

### 2.2. Search Strategy

Eight digital databases were searched for RCTs, including PubMed, Embase, Web of Science, Cochrane Library, China National Knowledge Infrastructure (CNKI), Chinese Science and Technology Periodical Database (VIP), Wanfang database, and China Biomedical Literature Service System (SinoMed). We identified articles published from initiation to September 2020. MeSH terms and free text terms are searched below: acupuncture therapy; acupuncture; electroacupuncture; transcutaneous electrical nerve stimulation; Acupoint embedding and acupoint catgut embedding; acupoint therapy. The MEDLINE search strategy is given in Supplementary File: Appendix 1.

### 2.3. Study Selection and Data Extraction

Two researchers independently carried out the search, selected the qualified literature according to the inclusion and exclusion criteria, and discussed with a third party if there was a disagreement. According to the data extraction table, the basic data, research characteristics, effect variables, and other content were extracted from the final included original documents.

### 2.4. Assessment of RCTs Quality

The Cochrane bias risk assessment tool is used for evaluation: (1) random method; (2) allocation hiding; (3) whether blinding is appropriate for the research object; (4) whether blinding is implemented for intervention implementers and outcome measurers; (5) completeness of the result data , lost to follow-up/withdrawal and intent (ITT) analysis; (6) selective reporting of research results, whether to report on adverse reactions, monitoring ovulation ultrasound, and other results; (7) other sources of bias: including funding sources. The two researchers conduct independent evaluations and discuss and resolve differences with a third party.

### 2.5. Statistical Analysis

Meta-analysis was performed using RevMan5.3 software of Cochrane Collaboration. The enumeration data used the risk ratio (RR) to express the combined effect size, while the measurement data used the mean difference (MD) or standard mean difference (SMD) to express, and each effect size was expressed with a 95% confidence interval (CI). The forest map performed statistical analysis of the effect size. To judge the rationality of the analysis of the combined studies, the chi-square test is used for heterogeneity analysis. When *P* > 0.1 and *I*^2^ <50%, which means that multiple similar studies have homogeneity, the fixed effects model would be used, and the random effects model would be applied if not. Sensitivity analysis and funnel plots will be generated when the included studies are enough (more than 10 RCTs) and thus explore the potential reasons for heterogeneity and bias.

## 3. Results

### 3.1. Study Selection

**Figure 1:**
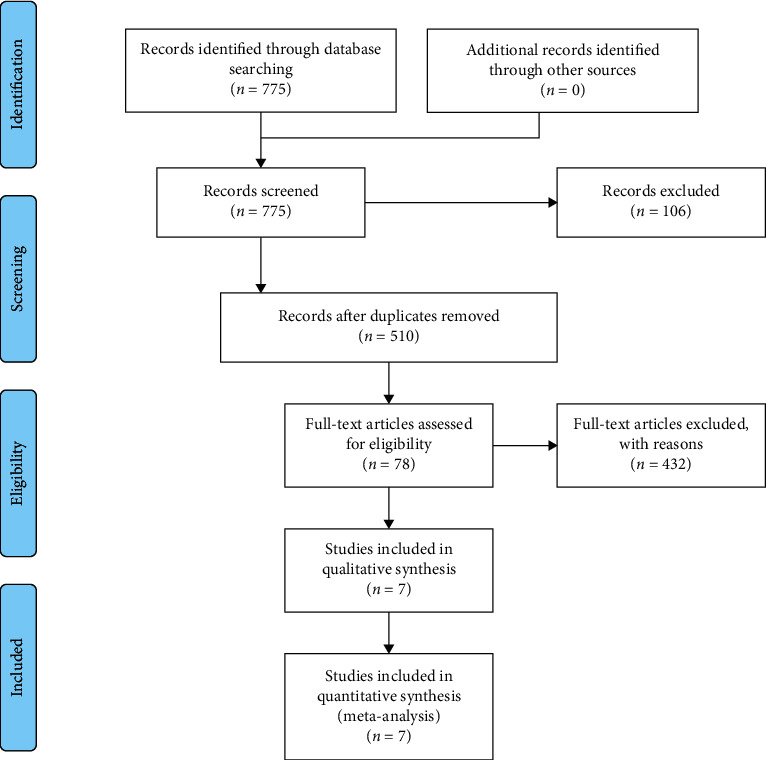
Flowchart of article screening process.

According to the search strategy, two researchers retrieved 775 studies in total, including 156 Chinese documents and 619 English documents. 251 documents were removed by machine checking, and 14 documents in the remaining documents were similar that had a possibility of duplicate publication and were excluded. According to the inclusion and exclusion criteria, 432 unqualified documents were eliminated by reading the titles and abstracts. A total of 78 documents were read and screened in full. The excluded documents included the following reasons: (1) combined nonacupuncture treatment; (2) self-controlled trials; (3) not taking clinical pregnancy as the outcome indicator; (4) having unclear diagnostic criteria. A total of 7 articles were finally included into the meta-analysis (Figure 1).

### 3.2. Study Selection

The number of patients included among the seven included articles [12–18] was 756, including 336 in the control group and 420 in the control group. The experimental group was treated with acupuncture or TEAS (transcutaneous electrical acupuncture stimulation), and the control group was divided into sham acupuncture and blank control. Both groups were prepared for pretransplantation and posttransplantation corpus luteum support to some extent. The two groups in each study shared the same course of treatment but not all consistent outcome indicators (Table 1). Five studies [12–14, 16, 18] included patients suffering RIF due to poor endometrial receptivity, while two studies [15, 17] included patients being infertile for unknown reasons. Five studies [12, 14–16, 18] took three times or more ET without pregnancy as the diagnostic criteria, while one study [17] used more than two times of ET, and one study [13] did not specify the number of ET failures. All studies described acupuncture points for treatment in detail. It could be found that the selected acupoints were mainly Zhongwan (RN12), Tianshu (ST25), Qihai (RN6), Guanyuan (RN4), Zhongji (RN3), and Zigong (EX-CA1). They are located in the abdomen, and the meridians of them are all connected to the Ren Vessel with the effect of drawing qi to its origin.

### 3.3. Risk of Bias Assessment of the Included Studies

**Figure 2:**
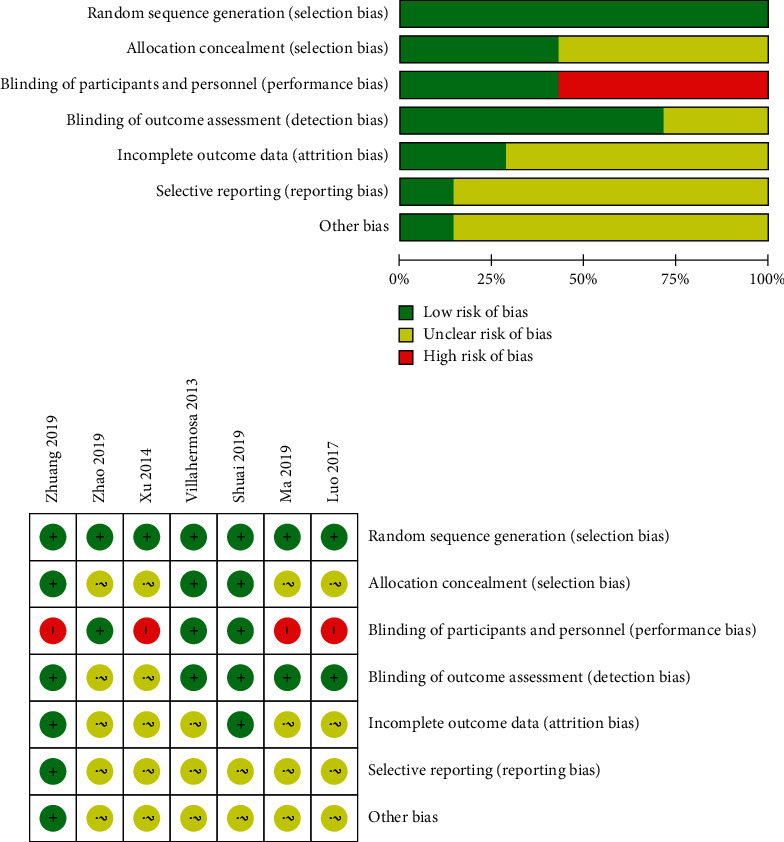
Risk of bias assessment.

As shown in Figure 2, the seven studies included in this meta-analysis are all randomized controlled trials, and all mention randomized allocation, of which five studies [12, 13, 16–18] explain the randomization methods in detail and three studies [16–18] mention the modus of allocation concealment. Among three studies [14, 17, 18], participants were blinded to sham acupuncture, but all studies could not conduct double-blind acupuncture. Two studies [16, 18] clearly point out the number and reasons for the loss of patients. All the literatures have not established enough clinical follow-up periods to observe long-term effects. Two articles [16, 18] underline that there is no selective reporting or publication bias in their studies. All the literatures indicate that there is no significant difference in initial value between groups, and the baseline data of the literature is well balanced (Supplementary File: Appendix 2-3). Other unknown biases are not clear.

### 3.4. Meta-Analysis

#### 3.4.1. Pregnancy Rate

**Figure 3:**
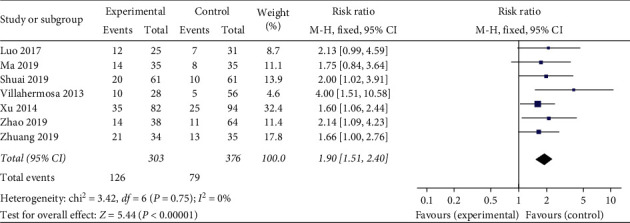
Effect of intervention on clinical pregnancy rate.

*Clinical Pregnancy Rate.* A total of seven studies used clinical pregnancy rate as an evaluation indicator. The statistical results demonstrate homogeneous (heterogeneity test, *X*^2^ = 3.42, *P* < 0.1, *I*^2^ = 0%). The pooled results showed that acupuncture treatment was prior to the sham/placebo or nonacupuncture group, with Relative Risk (RR) of 1.50 (95% Confidence Interval, 1.51 to 2.40; *P* < 0.05). There is statistical significance in clinical pregnancy rate between the two groups (Figure 3).

**Figure 4:**
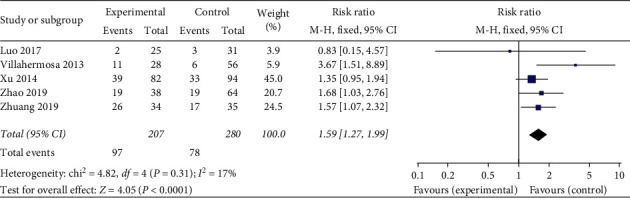
Effect of intervention on biochemical pregnancy rate.

*Biochemical Pregnancy Rate.* A total of five studies used biochemical pregnancy rate as an evaluation indicator. The statistical results demonstrate homogeneous (heterogeneity test, *X*^2^ = 4.82, *P* > 0.1, *I*^2^ = 17%). The pooled results showed that acupuncture treatment was prior to the sham/placebo or nonacupuncture group, with Relative Risk of 1.59 (95% Confidence Interval, 1.27 to 1.99; *P* < 0.05). There is statistical significance in the biochemical pregnancy rate between the two groups (Figure 4).

#### 3.4.2. Embryo

**Figure 5:**
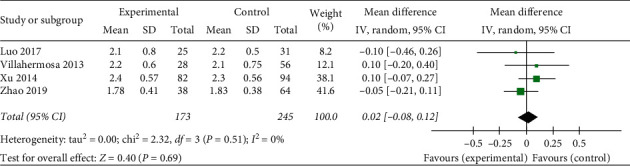
Effect of intervention on number of embryo transfers.

*Number of Embryo Transfers.* A total of six studies used the number of embryo transfers as an evaluation indicator. The statistical results demonstrate homogeneous (heterogeneity test, *X*^2^ = 2.32, *P* > 0.1, *I*^2^ = 0%). The pooled results showed that there is no statistical significance in number of embryo transfers among the acupuncture treatment group, sham/placebo or nonacupuncture group, with a mean difference (MD) of 0.02 (95% Confidence Interval, −0.08 to 0.12; *P* < 0.05) (Figure 5).

**Figure 6:**
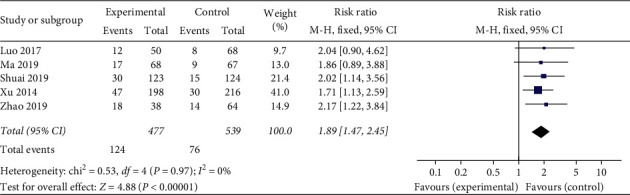
Effect of intervention on embryo implantation rate.

*Embryo Implantation Rate.* A total of five studies used embryo implantation rate as an evaluation indicator. The statistical results demonstrate homogeneous (heterogeneity test, *X*^2^ = 0.53, *P* > 0.1, *I*^2^ = 0%). The pooled results showed that acupuncture treatment was prior to the sham/placebo or non-acupuncture group, with Relative Risk of 1.89 (95% Confidence Interval, 1.47 to 2.45; *P* < 0.05). There is statistical significance in embryo implantation rate between the two groups, which is largely related to the clinical pregnancy rate (Figure 6).

#### 3.4.3. Endometrium

**Figure 7:**
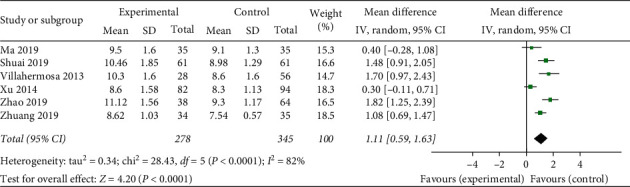
Effect of intervention on endometrium thickness.

*Endometrium Thickness.* A total of six studies used endometrial thickness as an evaluation indicator. The statistical results demonstrate heterogeneous (heterogeneity test, *X*^2^ = 28.43, *P* < 0.1, *I*^2^ = 82%). The pooled results showed that acupuncture treatment was prior to the sham/placebo or nonacupuncture group, with a mean difference of 1.11 (95% Confidence Interval, 0.59 to 1.63; *P* < 0.05). There is statistical significance in endometrial thickness between the two groups, but data are needed to be analyzed under the subgroup to determine the origin of heterogeneity (Figure 7).

**Figure 8:**
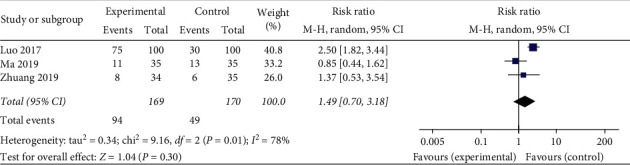
Effect of intervention on endometrium type.

*Endometrium Type.* A total of three studies used Type A endometrium as an evaluation indicator. The statistical results demonstrate heterogeneous (heterogeneity test, *X*^2^ = 9.16, *P* < 0.1, *I*^2^ = 78%). The pooled results showed that there is no statistical significance in Type A endometrium among acupuncture treatment group, sham/placebo, or nonacupuncture group, with Relative Risk of 1.49 (95% Confidence Interval, 0.70 to 3.18; *P* < 0.05). However, since the included studies and number of cases were not enough to support the results, we consider that this analysis is less referenced (Figure 8).

#### 3.4.4. Subgroup Analysis

**Figure 9:**
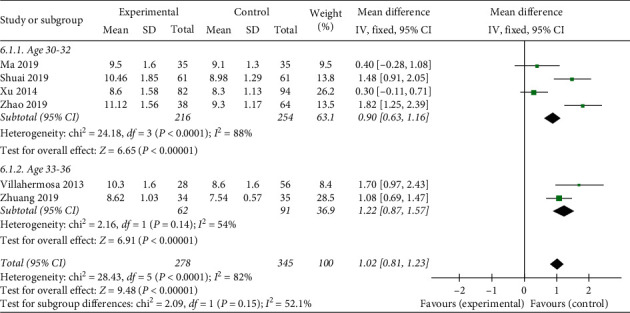
Subgroup of age on heterogeneity analysis.

**Figure 10:**
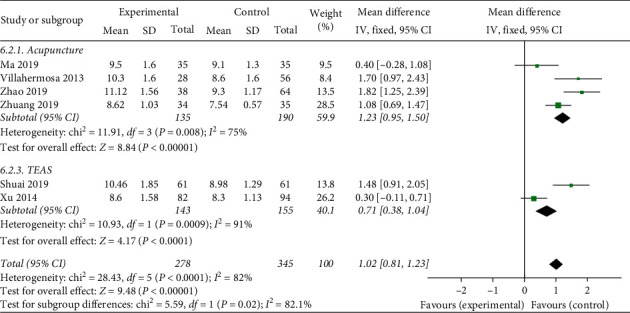
Subgroup of interventions with heterogeneity analysis.

The heterogeneity of the comparison of endometrial thickness through the analysis above could be found (*X*^2^ = 28.43, *P* < 0.1, *I*^2^ = 82%). To investigate the reason for it, we conducted two subgroup analysis according to the age and intervention of the studies. Unexpectedly, the subgroup analysis turned out to have no significant decrease in the heterogeneity (Figures 9 and 10). The results showed that neither age nor intervention contributed to the heterogeneity. For one reason, this negative result may be owing to the extraction of source which may be relative to other detection means, but it still lacks identification due to insufficient case information. For another reason, endometrial thickness may have been less relevant to endometrial receptivity and clinical pregnancy rate, so the outcomes differ from various studies, which causes heterogeneity.

### 3.5. Safety Analysis

One study [16] has reported the adverse reactions that three patients in the treatment group showed subcutaneous congestion at acupuncture points. Another two studies [15, 17] mentioned that no adverse reactions occurred. And the rest of the research did not mention whether adverse events happened or not during the treatment process. It can be considered that acupuncture as an interventional treatment in IVF cycles of recurrent implantation failure is safe.

### 3.6. Publication Bias

Since only seven articles were included, the funnel plot was not produced to evaluate the publication bias of the included articles.

## 4. Discussion

### 4.1. Main Finding

The aim of this analysis was to identify the efficacy and safety of acupoint stimulation therapy for RIF patients. The results of meta-analysis show that for the outcome measurements including clinical pregnancy rate, biochemical pregnancy rate, embryo implantation rate, and endometrial thickness, the use of acupuncture in the treatment group is higher than the sham or nonacupuncture group, in which the difference is statistically significant. These results are believable and reliable. The included studies are highly homogeneous and there is no obvious publication bias. However, large heterogeneity appears in the statistical analysis of the endometrial thickness. Moreover, there was no statistical significance in the number of embryo transfers and the type of endometrium.

### 4.2. Interpretation

In this meta-analysis, there is large heterogeneity in the statistical analysis of the endometrial thickness. The heterogeneity has not been explained in the subgroup analysis as we failed to determine the reasons for it. It also meets the current discussion of whether endometrial thickness is related to endometrial receptivity or pregnancy rate. The answer of this question is still lack of direct and sufficient evidence to support. An article [19] investigating the influence of acupuncture on endometrial receptivity in Chinese women in the past ten years summarized that the mechanism of acupuncture to improve endometrial receptivity is to improve the morphology of the endometrium [20–22], to promote endometrial microcirculation [23, 24], to regulate estrogen and progesterone and its receptors bidirectionally [25], and to adjust the expression of integrin *α*v*β*3, LIF, VEGF, HOXA10, and other molecular biological regulatory factors [26–30], but the impact of acupuncture on the thickness of the endometrium has not been mentioned yet. The latest meta-analysis for evaluating endometrial receptivity markers [31] suggested that, in the randomized controlled trials of comparing endometrial thickness of the pregnant group and the nonpregnant group, there is no significant difference was observed in the endometrial thickness measured on the day of IUI or IVF between the groups, so the article assume that endometrial thickness may not be associated with clinical pregnancy or be a useful test for endometrial receptivity. There are articles [32] that believed that the thickness of the endometrium has little effect on the pregnancy rate, but extreme condition of endometrium like too thin or too thick seems not conducive to embryo implantation [33, 34]. Nevertheless, some articles [35, 36], through clinical observation and randomized controlled trials, found that endometrial thickness during fresh IVF cycles was a better predictor of endometrial receptivity than other indicator. There is also an article [37] that believed that it is not the thickness of the endometrium but the volume of the endometrium that affects the endometrium receptivity. This study found that endometrial volume of <2 ml on the day of embryo transfer was a better predictor for low endometrial receptivity than endometrial thickness on the same day and resulted in significant lower IVF clinical pregnancy and implantation rates. Therefore, the question of whether the endometrial thickness can be used as one of the criteria for evaluating endometrial receptivity needs further experiments to verify.

### 4.3. Limitation and Prospect

The seven studies included in this meta-analysis are all randomized controlled trial research documents. There is no significant difference in the baseline data between groups. However, this analysis also demonstrates the limitations of current clinical randomized controlled trials. The methodological quality evaluation of the included researches shows that there are most of the clinical studies that have methodological problems such as failing to apply double-blindness, lack of allocation concealment, and no follow-up. Moreover, the absence of reporting sample size, calculation methods, or usage analysis can lead to the risk of bias, which affects the accuracy and reliability of the test results to a certain extent. Therefore, we look forward to more large-scale, multicenter, large-sample, and double-blind randomized controlled trials in the future. At the same time, using strict allocation concealment and other research design plans, setting specific evaluation criteria of adverse reactions, periodical follow-up, and standardizing proper treatment for the control group are intensively needed. Apart from that, clinically meaningful observation indicators such as the high qualified embryo rate and health economics evaluation should be included to ensure the scientificity, objectivity, and reliability of the research conclusions. Therefore, it will make the acupuncture intervention treatment more normalized for RIF at home and abroad. At the same time, it is necessary to develop further researches on the mechanism of acupuncture and moxibustion treatments, and to seek the connection of acupuncture and RIF on the molecular level. New ways are waiting to integrate and develop the merits of treatment of Chinese and Western medicine, reduce side effects, and improve clinical pregnancy rate.

## Figures and Tables

**Table 1:** Characteristics of included studies.

Author, year	Sample size	Excluded	RIF reason	Age (mean)		Acupuncture group	Control group	IVF outcome
				Acupuncture group	Control group			
Ma 2019	70	0	ER	30.04 ± 2.98	30.55 ± 3.71	MA + HRT	HRT	CPR EIR ET EM EBF SDS ETN
Luo 2017	56	0	ER	33.8 ± 4.6	32.5 ± 4.1	MA + moxibustion + levofloxacin + dydrogesterone	Levofloxacin + dydrogesterone	CPR ET EIR ETN HQE MR BPR EM
Zhao 2019	102	0	ER	32.57 ± 4.25	32.95 ± 4.37	EA + TDP + HRT	Sham EA + HRT/HRT	CPR ET ETN SHL EIR BPR
Xu 2014	176	0	Unknown	32.5 ± 4.6	31.9 ± 4.3	TEAS + HRT	HRT	CPR SHL AF BMI ET BPR EIR ETN
Zhuang 2019	72	3	ER	34.29 ± 5.31	34.26 ± 5.30	MA + FET	FET	CPR ET EM EBF (PI; RI) BPR LBR TCMS
Villaherm-osa 2013	84	0	Unknown	36.0 ± 2.7	36.3 ± 2.15	MA + moxibustion	Sham MA/blank	CPR ON ET ETN BPR
Shuai 2019	124	2	ER	31.23 ± 3.78	31.58 ± 3.07	TEAS	MTEAS	CPR LBR ETN ET E2 EIR ON

EA: electro acupuncture, MA: manual acupuncture, TEAS: transcutaneous electrical acupuncture stimulation, MTEAS: mock transcutaneous electrical acupuncture stimulation, FET: frozen embryo transplantation, HRT: hormone replacement therapy, ER: endometrial receptivity, BPR: biochemical pregnancy rate, CPR: clinical pregnancy rate, BPR: biochemical pregnancy rate, MR: miscarriage rate, LBR: live birth rate, ETN: embryos transfer number, EIR: embryos implantation rate, MR: miscarriage rate, BMI: body mass index, ET: endometrial thickness, EM: endometrial morphology, EBF: endometrial blood flow, RI: resistance index, PI: pulse index, AF: antral follicles, HQE: high-quality embryo, SHL: sex hormone levels, ON: oocyte number, TCMS: traditional Chinese medicine syndrome, and SDS: self-rating depression scale.

## Data Availability

The data used to support the study were used from the references [12–18].

## Supplementary Materials

Supplementary Materials Appendix 1: the medline search strategy. Appendix 2: a summary of mean, standard difference, and sample size between groups for included studies in the meta-analysis. Appendix 3: consistency test.
