# Supplementary material for: The Effects of Acupuncture on Pregnancy Outcomes of Recurrent Implantation Failure: A Systematic Review and Meta-Analysis
Source: Evid Based Complement Alternat Med. 2021 Feb 2;2021:6661235. doi: 10.1155/2021/6661235 (PMC7878089; doi:10.1155/2021/6661235)
Supplement: Supplementary Materials — Appendix 1: the medline search strategy. Appendix 2: a summary of mean, standard difference, and sample size between groups for included studies in the meta-analysis. Appendix 3: consistency test. [file 6661235.f1.docx]

**Appendix 1: Search Strategies.**

**PubMed**

("Acupuncture"[MeSH Terms] OR "Acupuncture Therapy"[MeSH Terms] OR "acupuncture, ear"[MeSH Terms] OR "Electroacupuncture"[MeSH Terms] OR "Moxibustion"[MeSH Terms] OR "Acupuncture"[Title/Abstract] OR "Electroacupuncture"[Title/Abstract] OR "acustimulation"[Title/Abstract] OR "electro-acupuncture"[Title/Abstract] OR "needling"[Title/Abstract] OR "needle pricking"[Title/Abstract] OR "fire needle"[Title/Abstract] OR "point application"[Title/Abstract] OR "three edged needle"[Title/Abstract] OR "Moxibustion"[Title/Abstract] OR "auricular therapy"[Title/Abstract] OR (("ear"[MeSH Terms] OR "ear"[All Fields]) AND "point therapy"[Title/Abstract]) OR "moxabustion"[Title/Abstract] OR "point injection"[Title/Abstract] OR "transcutaneous electrical acupoint stimulation"[Title/Abstract] OR "TEAS"[Title/Abstract] OR "acupoint embedding"[Title/Abstract] OR "acupoint catgut embedding"[Title/Abstract]) AND ("repeated implantation failure"[Title/Abstract] OR "RIF"[Title/Abstract] OR "assisted reproductive technology failure"[Title/Abstract] OR "embryo transfer failure"[Title/Abstract] OR "in vitro fertilization embryo transfer failure"[Title/Abstract] OR "ivf et failure"[Title/Abstract] OR "recurrent implantation failures"[Title/Abstract] OR "IVF-ET"[Title/Abstract] OR "ICSI"[Title/Abstract] OR "intracytoplasmic sperm injection"[Title/Abstract] OR "ART"[Title/Abstract] OR "assisted reproductive technology"[Title/Abstract] OR "fertilization in vitro"[Title/Abstract] OR "plant preparation"[Title/Abstract] OR "embryo implantation"[Title/Abstract]) AND (("randomized controlled trial"[Publication Type] OR "controlled clinical trial"[Publication Type] OR "randomized"[Title/Abstract] OR "placebo"[Title/Abstract] OR "clinical trials as topic"[MeSH Terms:noexp] OR "randomly"[Title/Abstract] OR "trial"[Title]) NOT ("animals"[MeSH Terms] NOT ("humans"[MeSH Terms] AND "animals"[MeSH Terms])))

#1 "Acupuncture"[Mesh] OR "Acupuncture Therapy"[Mesh] OR "Acupuncture, Ear"[Mesh] OR "Electroacupuncture"[Mesh] OR "Moxibustion"[Mesh] OR acupuncture[Title/Abstract] OR electroacupuncture[Title/Abstract] OR acustimulation[Title/Abstract] OR electro-acupuncture[Title/Abstract] OR needling[Title/Abstract] OR needle pricking[Title/Abstract] OR fire needle[Title/Abstract] OR Point application[Title/Abstract] OR three-edged needle[Title/Abstract] OR moxibustion[Title/Abstract] OR auricular therapy[Title/Abstract] OR ear point therapy[Title/Abstract] OR moxabustion[Title/Abstract] OR point injection[Title/Abstract] OR transcutaneous electrical acupoint stimulation[Title/Abstract] OR TEAS[Title/Abstract] OR acupoint embedding[Title/Abstract] OR acupoint catgut embedding[Title/Abstract] OR catgut embedding[Title/Abstract] OR embedding thread[Title/Abstract]

#2 repeated implantation failure[Title/Abstract] OR RIF[Title/Abstract] OR assisted reproductive technology failure[Title/Abstract] OR embryo transfer failure[Title/Abstract] OR In Vitro Fertilization-Embryo Transfer failure[Title/Abstract] OR IVF-ET failure[Title/Abstract] OR recurrent implantation failures[Title/Abstract] OR IVF-ET[Title/Abstract] OR ICSI[Title/Abstract] OR Intracytoplasmic sperm injection[Title/Abstract] OR ART[Title/Abstract] OR assisted reproductive technology[Title/Abstract] OR Fertilization in Vitro[Title/Abstract] OR plant preparation[Title/Abstract] OR embryo implantation[Title/Abstract] OR in Vitro Fertilization [Title/Abstract]

#3 (randomized controlled trial[pt] OR controlled clinical trial[pt] OR randomized[tiab] OR placebo[tiab] OR clinical trials as topic[mesh:noexp] OR randomly[tiab] OR trial[ti]) NOT (animals [mh] NOT (humans [mh] AND animals[mh]))

#4 #1 AND #2 AND #3

**EMBASE:**

('acupuncture'/exp OR 'electroacupuncture'/exp OR 'moxibustion'/exp OR acupuncture:ab,ti OR electroacupuncture:ab,ti OR acustimulation:ab,ti OR 'electro acupuncture':ab,ti OR needling:ab,ti OR 'needle pricking':ab,ti OR 'fire needle':ab,ti OR 'point application':ab,ti OR 'three-edged needle':ab,ti OR moxibustion:ab,ti OR moxabustion:ab,ti OR 'auricular therapy':ab,ti OR 'ear point therapy':ab,ti OR 'transcutaneous electrical acupoint stimulation':ab,ti OR 'teas':ab,ti OR 'acupoint embedding':ab,ti OR 'acupoint catgut embedding':ab,ti) AND ('repeated implantation failure':ab,ti OR rif:ab,ti OR 'assisted reproductive technology failure':ab,ti OR 'embryo transfer failure':ab,ti OR 'in vitro fertilization-embryo transfer failure':ab,ti OR 'ivf-et failure':ab,ti OR 'recurrent implantation failures':ab,ti OR 'ivf-et':ab,ti OR 'icsi':ab,ti OR 'intracytoplasmic sperm injection':ab,ti OR 'art':ab,ti OR 'assisted reproductive technology':ab,ti OR 'fertilization in vitro':ab,ti OR 'plant preparation':ab,ti OR 'embryo implantation':ab,ti) AND ('crossover procedure':de OR 'double-blind procedure':de OR 'randomized controlled trial':de OR 'single-blind procedure':de OR random*:de,ab,ti OR factorial*:de,ab,ti OR crossover*:de,ab,ti OR ((cross NEXT/1 over*):de,ab,ti) OR placebo*:de,ab,ti OR ((doubl* NEAR/1 blind*):de,ab,ti) OR ((singl* NEAR/1 blind*):de,ab,ti) OR assign*:de,ab,ti OR allocat*:de,ab,ti OR volunteer*:de,ab,ti)

#1 'acupuncture'/exp OR 'electroacupuncture'/exp OR 'moxibustion'/exp OR acupuncture:ab,ti OR electroacupuncture:ab,ti OR acustimulation:ab,ti OR 'electro acupuncture':ab,ti OR needling:ab,ti OR 'needle pricking':ab,ti OR 'fire needle':ab,ti OR 'point application':ab,ti OR 'three-edged needle':ab,ti OR moxibustion:ab,ti OR moxabustion:ab,ti OR 'auricular therapy':ab,ti OR 'ear point therapy':ab,ti OR 'transcutaneous electrical acupoint stimulation':ab,ti OR 'teas':ab,ti OR 'acupoint embedding':ab,ti OR 'acupoint catgut embedding':ab,ti OR 'catgut embedding':ab,ti OR 'embedding thread':ab,ti

#2 'repeated implantation failure':ab,ti OR rif:ab,ti OR 'assisted reproductive technology failure':ab,ti OR 'embryo transfer failure':ab,ti OR 'in vitro fertilization-embryo transfer failure':ab,ti OR 'ivf-et failure':ab,ti OR 'recurrent implantation failures':ab,ti OR 'ivf-et':ab,ti OR 'icsi':ab,ti OR 'intracytoplasmic sperm injection':ab,ti OR 'art':ab,ti OR 'assisted reproductive technology':ab,ti OR 'fertilization in vitro':ab,ti OR 'plant preparation':ab,ti OR 'embryo implantation':ab,ti OR 'in vitro fertilization':ab,ti

#3 'crossover procedure':de OR 'double-blind procedure':de OR 'randomized controlled trial':de OR 'single-blind procedure':de OR random*:de,ab,ti OR factorial*:de,ab,ti OR crossover*:de,ab,ti OR ((cross NEXT/1 over*):de,ab,ti) OR placebo*:de,ab,ti OR ((doubl* NEAR/1 blind*):de,ab,ti) OR ((singl* NEAR/1 blind*):de,ab,ti) OR assign*:de,ab,ti OR allocat*:de,ab,ti OR volunteer*:de,ab,ti

#4 #1 AND #2 AND #3

**Cochrane Library**

#1 MeSH descriptor: [Acupuncture] explode all trees

#2 MeSH descriptor: [Acupuncture Therapy] explode all trees

#3 MeSH descriptor: [Acupuncture, Ear] explode all trees

#4 MeSH descriptor: [Electroacupuncture] explode all trees

#5 MeSH descriptor: [Moxibustion] explode all trees

#6 acupuncture:ab,ti OR electroacupuncture:ab,ti OR acustimulation:ab,ti OR 'electro acupuncture':ab,ti OR needling:ab,ti OR 'needle pricking':ab,ti OR 'fire needle':ab,ti OR 'point application':ab,ti OR 'three-edged needle':ab,ti OR moxibustion:ab,ti OR moxabustion:ab,ti OR 'auricular therapy':ab,ti OR 'ear point therapy':ab,ti OR ‘transcutaneous electrical acupoint stimulation’:ab,ti OR ‘TEAS’:ab,ti OR ‘acupoint embedding’:ab,ti OR ‘acupoint catgut embedding’:ab,ti OR ‘catgut embedding’:ab,ti OR ‘embedding thread’:ab,ti

#7 #1 OR #2 OR #3 OR #4 OR #5 OR #6

#8 'repeated implantation failure':ab,ti OR rif:ab,ti OR 'assisted reproductive technology failure':ab,ti OR 'embryo transfer failure':ab,ti OR 'in vitro fertilization-embryo transfer failure':ab,ti OR 'ivf-et failure':ab,ti OR 'recurrent implantation failures':ab,ti OR ‘IVF-ET’:ab,ti OR ‘ICSI’:ab,ti OR ‘Intracytoplasmic sperm injection’:ab,ti OR ‘ART’:ab,ti OR ‘assisted reproductive technology’:ab,ti OR ‘Fertilization in Vitro’:ab,ti OR ‘plant preparation’:ab,ti OR ‘embryo implantation’:ab,ti OR ‘in vitro fertilization’:ab,ti

#9 #7 AND #8

**Web of Science**

#1

TS=(acupuncture OR electroacupuncture OR acustimulation OR electro-acupuncture OR needling OR needle pricking OR fire needle OR Point application OR three-edged needle OR moxibustion OR ear point therapy OR moxabustion OR point injection OR transcutaneous electrical acupoint stimulation OR TEAS OR acupoint embedding OR acupoint catgut embedding OR embedding thread OR catgut embedding)

#2 TS=(repeated implantation failure OR RIF OR assisted reproductive technology failure OR embryo transfer failure OR In Vitro Fertilization-Embryo Transfer failure OR IVF-ET failure OR recurrent implantation failures OR IVF-ET OR ICSI OR Intracytoplasmic sperm injection OR ART OR assisted reproductive technology OR Fertilization in Vitro OR plant preparation OR embryo implantation OR recurrent implantation failures OR in vitro fertilization）

#3 TS=(randomized controlled trial OR controlled clinical trial OR randomized OR placebo OR clinical trials OR randomly OR trial)

#4

#3 AND #2 AND #1

**Appendix 2:** **A summary of mean, standard difference, and sample size between groups for included studies in the meta-analysis**

| Study | Treatment | Age | Duration of infertility | N |
| --- | --- | --- | --- | --- |
|  |  | Mean±sd | Mean±sd |  |
| Ma 2019 | MA+HRT | 30.04±2.98 | 4.4±1.8 | 35 |
|  | HRT | 30.55±3.71 | 4.9±1.5 | 35 |
| Luo 2017 | MA+  Moxibustion + Levofloxacin+ Dydrogesterone | 33.8±4.6 | 4.5±2.6 | 25 |
|  | Levofloxacin+ Dydrogesterone | 32.5±4.1 | 4.6±2.3 | 31 |
| Zhao 2019 | EA+TDP+ HRT | 32.57±4.25 | 6.11±1.62 | 38 |
|  | Sham EA+ HRT | 33.71±4.22 | 6.40±1.31 | 34 |
|  | HRT | 32.10±4.54 | 5.30±1.75 | 30 |
| Xu 2014 | TEAS+HRT | 32.5±4.6 | 5.1±3.7 | 82 |
|  | HRT | 31.9±4.3 | 4.8±3.6 | 94 |
| Zhuang 2019 | MA+FET | 34.29±5.31 | 4.38±1.60 | 34 |
|  | FET | 34.26±5.30 | 4.20±1.94 | 35 |
| Villahermosa 2013 | MA+Moxibustion | 36.0±2.7 | 4.4±1.5 | 28 |
|  | Sham MA | 36.2±2.2 | 4.3±1.4 | 28 |
|  | Blank | 36.4±2.1 | 4.7±1.9 | 28 |
| Shuai 2019 | TEAS | 31.23±3.78 | 5.09±2.78 | 61 |
|  | MTEAS | 31.58±3.07 | 5.71±3.59 | 61 |

*Abbreviation. EA electro acupuncture, MA manual acupuncture, TEAS transcutaneous electrical acupuncture stimulation, MTEAS mock transcutaneous electrical acupuncture stimulation, FET* *Frozen embryo transplantation, HRT hormone replacement therapy.*

**Appendix 3: Consistency test.**


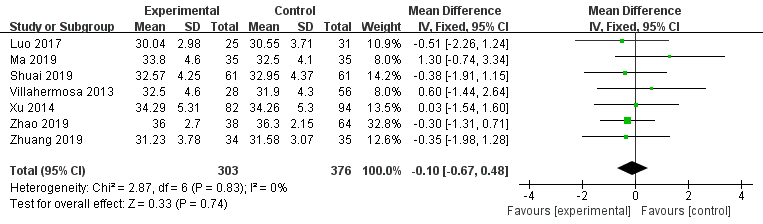


*Figure1. Age (P>0.1, no significant different in AGE)*


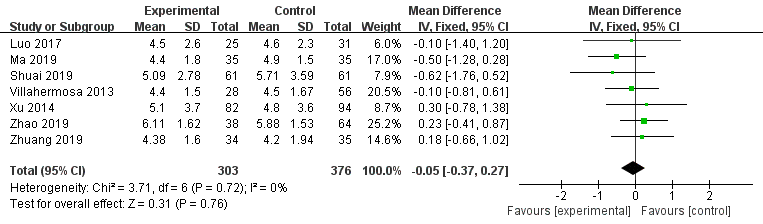


*Figure2. Duration of infertility (P>0.1, no significant different in Duration of infertility)*
